# Shear Behavior of Concrete Beams Reinforced with a New Type of Glass Fiber Reinforced Polymer Reinforcement: Experimental Study

**DOI:** 10.3390/ma13051159

**Published:** 2020-03-05

**Authors:** Czesław Bywalski, Michał Drzazga, Maciej Kaźmierowski, Mieczysław Kamiński

**Affiliations:** Faculty of Civil Engineering, Wroclaw University of Science and Technology, Wybrzeże Wyspiańskiego 27, 50-370 Wrocław, Poland; michal.drzazga@pwr.edu.pl (M.D.); maciej.kazmierowski@pwr.edu.pl (M.K.); mieczyslaw.kaminski@pwr.edu.pl (M.K.)

**Keywords:** GFRP, FRP reinforcement, shear, capacity, reinforced concrete beams

## Abstract

The article presents experimental tests of a new type of composite bar that has been used as shear reinforcement for concrete beams. In the case of shearing concrete beams reinforced with steel stirrups, according to the theory of plasticity, the plastic deformation of stirrups and stress redistribution in stirrups cut by a diagonal crack are permitted. Tensile composite reinforcement is characterized by linear-elastic behavior throughout the entire strength range. The most popular type of shear reinforcement is closed frame stirrups, and this type of Fiber Reinforced Polymer (FRP) shear reinforcement was the subject of research by other authors. In the case of FRP stirrups, rupture occurs rapidly without the shear reinforcement being able to redistribute stress. An attempt was made to introduce a quasi-plastic character into the mechanisms transferring shear by appropriately shaping the shear reinforcement. Experimental material tests covered the determination of the strength and deformability of straight Glass Fiber Reinforced Polymer (GFRP) bars and GFRP headed bars. Experimental studies of shear reinforced beams with GFRP stirrups and GFRP headed bars were carried out. This allowed a direct comparison of the shear behavior of beams reinforced with standard GFRP stirrups and a new type of shear reinforcement: GFRP headed bars. Experimental studies demonstrated that GFRP headed bars could be used as shear reinforcement in concrete beams. Unlike GFRP stirrups, these bars allow stress redistribution in bars cut by a diagonal crack.

## 1. Introduction

Corrosion of reinforcing steel is the main factor causing material degradation in reinforced concrete structures, which, if exposed to particularly adverse environmental conditions, cease to meet the requirements of durability and reliability in a facility in a relatively short time [1,2,3,4]. The durability of reinforced concrete structures is particularly important for industrial facilities [5,6,7]. The use of non-metallic reinforcement as the main reinforcement of concrete elements is one way to exclude corrosion and thus extend the service life of the structure. Reinforced concrete elements embedded in structures exposed to particularly adverse weather and operating conditions require time-consuming and cost-intensive renovation. There is growing interest in using non-corrosive Fiber Reinforced Polymer (FRP) reinforcement, especially in engineering structures that will be exposed to harmful effects for a long period of use [2,6,8,9,10]. Among types of bar-shaped polymer reinforcement, we distinguish bars made of glass fiber (Glass Fiber Reinforced Polymer (GFRP)), carbon fiber (Carbon Glass Fiber Reinforced Polymer (CFRP)), basalt fiber (Basalt Glass Fiber Reinforced Polymer (BFRP)), and aramid fiber (Aramid Glass Fiber Reinforced Polymer (AFRP)). FRP bars are made of continuous fibers impregnated with polymeric resins. Continuous fibers with high stiffness and high strength are embedded in and bonded together by the polymeric matrix with low stiffness. In the FRP composites, the reinforcing fibers are the core, which determine the stiffness and strength of the material in the direction of the fibers. FRP composite bars are marked by good mechanical (high tensile strength) and physical properties (much lower density than reinforcing steel) [11]. FRP bars have been used in concrete elements in facilities particularly exposed to aggressive environments and in facilities whose proper functioning is dependent, among other things, on the electromagnetic neutrality of the construction elements. FRP composite bars are electromagnetically neutral and are therefore used in facilities requiring special precision of operation (no interference with devices operating inside the facility) and in infrastructure facilities (elimination of stray currents causing electro-corrosion) [4,11]. Composite reinforcements can be easily cut, which enables their effective use in temporary elements, e.g., as parts of tunnels [11].

Based on, among others, Fib Bulletin 40 [11] and ACI 440.1R-15 [12], the use of composite bars as longitudinal bending reinforcement has been much better researched than the use of these bars as transverse reinforcement. In order for the entire structural element to be corrosion-resistant and electromagnetically neutral, not only longitudinal reinforcement (flexural reinforcement), but also transverse reinforcement must be made of a suitable material. The most popular type of shear reinforcement is closed frame stirrups surrounding longitudinal reinforcement, put closest to the surface of the concrete element. This is why stirrups in reinforced concrete structures should be classified as the most vulnerable to corrosion. The above-mentioned factors have stimulated researchers to find a solution to the problem of material degradation due to corrosion by introducing transverse reinforcement in the form of frames made of polymer bars. Nagasaka et al. [13], Shehata [14], and Ahmed [15] indicated that direct replacement of steel stirrups with FRP stirrups is not possible due to differences resulting, for example, from mechanical properties. Polymer reinforcement is marked by high tensile strength, a relatively low modulus of elasticity (except CFRP), and linearly, elastic behavior in the entire strength range. Bentz et al. [16] indicated that the above also implies differences in the behavior of support zones reinforced longitudinally with FRP bars. Oller et al. [17] and Kosior-Kazberuk [18] indicated that, in accordance with the superposition principle, the shear load capacity of an element loaded with transverse force is dependent on the following factors: shear reinforcement contribution, load capacity resulting from aggregate interlocking, the load capacity of concrete in the compressed zone, the dowel action of longitudinal reinforcement, and the residual tensile strength of concrete across the crack. Composite reinforcement is characterized by a much lower modulus of elasticity than steel reinforcement. The distance from compressed fibers to the neutral axis in a concrete element reinforced longitudinally with FRP bars is smaller after cracking, than in the case of concrete elements reinforced with steel, the range of the compressed zone of the imported cross-section being smaller. This is due to the lower axial stiffness of FRP reinforcement. The range of the compression zone is smaller, which means that the shear capacity of concrete in the compression zone is also smaller. The crack opening width is larger in the case of FRP reinforcement [6,9,10,11], and the component related to the aggregate interlocking is reduced. The low transverse stiffness of FRP bars significantly reduces the component resulting from dowel action [12,17,19,20]. Assuming the same longitudinal reinforcement surface, a concrete element reinforced with FRP bars thus has a lower shear capacity than a concrete element reinforced with steel bars [21,22,23]. Ahmed [15], Kurth [24], and Jumaa et al. [25] indicated that a significant difference between an element reinforced with FRP bars and a concrete element reinforced with steel is a considerable reduction in the tensile strength of the bent FRP bar (stress concentration in the bent zone). This is why it is necessary to control the stresses in the stirrup bars to avoid rupture of the bent fragment. Another aspect is the lack of plasticity of composite reinforcement. Nagasaka et al. [13] and Razaqpur et al. [26] distinguished two failure modes of an FRP bar reinforced element subjected to shearing force: stirrup rupture and crushing of the compressed concrete. The rupture of composite bars is more fragile than crushing concrete and occurs when one of the cut stirrups reaches its tensile strength. Support zones reinforced with steel stirrups are characterized by a lack of stress redistribution ability.

Considering the above and the current state of knowledge reported in the literature, it should be stated that the behavior of support zones reinforced with FRP bars differs from those reinforced with steel. The limited number of studies on the subject does not permit explicit determination of the behavior of support zones reinforced with composite bars, especially support zones with low shear slenderness. Drzazga [27] indicated that while few research results are presented in the relevant literature concerning slender support zones, there are no results whatsoever available of research on short support zones reinforced transversally with GFRP bars, where the failure mechanism would be determined by the failure of transverse reinforcement (the shear–tension failure). Among others, Krall et al. [28], when testing deep beams, obtained only the failure in the form of crushing concrete without the GFRP stirrups’ rupture. No papers were found in which GFRP bars with head anchorage were used as shear reinforcement. The pull-out tests [29,30] of this type of anchorage indicated a more plastic nature of failure, if the bar slipped from the anchor head. In view of the shortage of research, this article presents the results of experimental tests of short shear zones reinforced with GFRP stirrups and GFRP headed bars where the failure mechanism is determined by the failure of transverse reinforcement. The most commonly used polymer bar reinforcement is GFRP reinforcement. This is due not only to the good properties of reinforcement made of glass fiber, but also to its relatively low price [11,31]. Therefore, the research program included this type of fiber. The article presents the results of experimental tests of GFRP bars and eight concrete beam supporting zones with differently shaped longitudinal (GFRP or steel) and transverse (GFRP stirrups or GFRP headed bars) reinforcement.

## 2. Experimental Program

**Figure 1:**
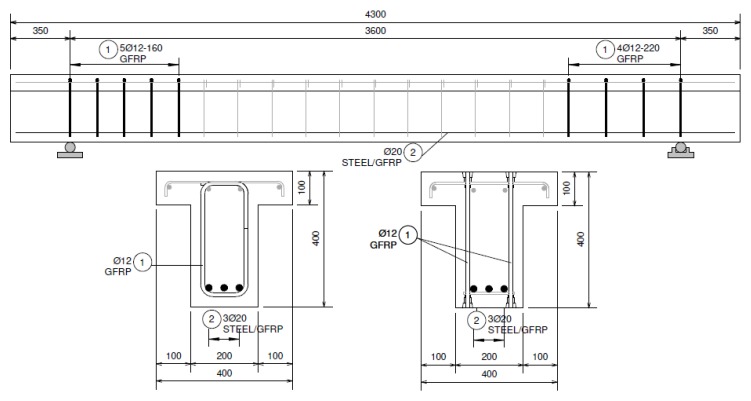
Reinforcement drawing of beams.

The main purpose of the experimental research was to verify the failure modes of beams reinforced with GFRP stirrups and GFRP headed bars. Four beams with differently shaped longitudinal and transverse reinforcement were tested. All beams were loaded with forces concentrated at a distance of 600 mm from the support (Figure 1). It was possible to verify the shear load capacity and failure modes of beams with low shear slenderness (*a*/*d* ratio ≈ 1.7 < 2.5).

### 2.1. Test Specimens

**Figure 2:**
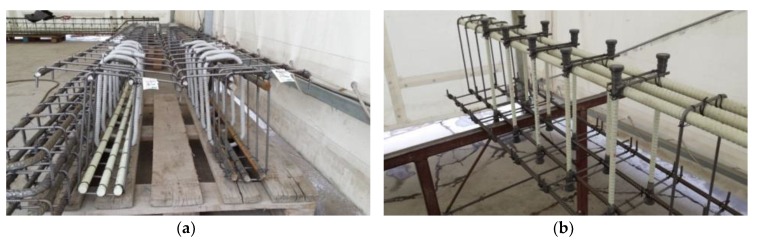
Reinforcement details of test specimens based on example beams: (**a**) B-1 and B-2; (**b**) B-3.

The beams were designed as T-sections with a 400 mm section height, 100 mm flange height, 400 mm flange width, and 200 mm web width. The total length of the beams was 4300 mm. The elements differed in the type of longitudinal reinforcement and the type of shear reinforcement. Twenty millimeter steel bars and 20 mm GFRP bars were used as longitudinal tensile reinforcement. GFRP stirrups with a diameter of 12 mm and GFRP headed bars with a diameter of 12 mm were used as shear reinforcement. Each beam was designed with two differently reinforced support zones; the same type of reinforcement was used throughout the entire beam, but the spacing of stirrups and headed bars was differentiated. The beams were designed so that the shear zones had the same area of tensioned bars taken into account in dowel action. A high class of concrete was used (concrete compressive strength around 70 MPa), and the T-section was used in order to increase the flexure strength. The method of reinforcing research elements is shown in Table 1 and Figure 1. Figure 2 presents the reinforcement cages of selected beams.

### 2.2. Material Properties

The beam testing was accompanied by the determination of the average compressive strength and the average modulus of elasticity of the concrete. The mechanical properties of the concrete were determined in cylindrical samples with a diameter of 150 mm and a height of 300 mm. The average compressive strength of the concrete for beams B-1, B-2, B-3, and B-4 was 68.94 MPa, 70.13 MPa, 65.47 MPa, and 71.39 MPa, respectively. The average modulus of the concrete elasticity under compression in the case of beams B-1, B-2, B-3, and B-4 was 35.47 GPa, 36.63 GPa, 35.97 GPa, and 38.07 GPa, respectively.

As longitudinal reinforcement, steel bars with a diameter of 20 mm and a characteristic yield strength of 500 MPa and GFRP bars with a diameter of 20 mm and an average tensile strength > 1100 MPa, as declared by the manufacturer, were used. The modulus of elasticity of the steel bars and GFRP bars was 200 GPa and 57 GPa, respectively, as declared by the manufacturer. GFRP stirrups with a diameter of 12 mm and headed bars with a diameter of 12 mm were used as shear reinforcement.

**Figure 3:**
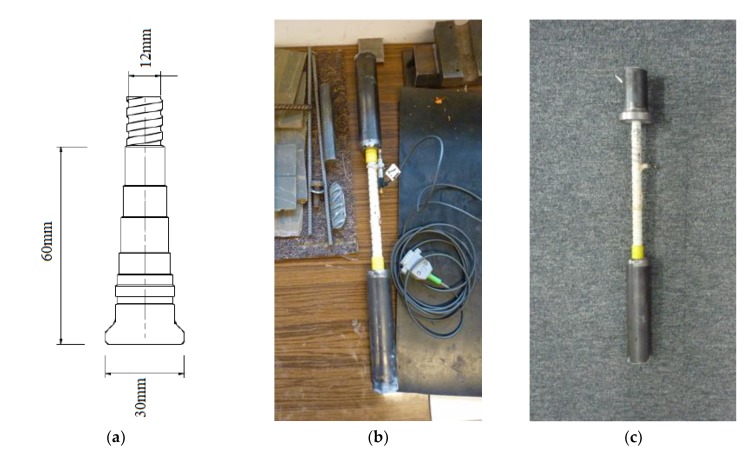
Samples for determining the tensile behavior of GFRP bars: (**a**) dimensions of the head anchorage of GFRP Schöck Combar; (**b**) straight bar sample; (**c**) headed bar sample.

Both stirrups and headed bars were a Schöck product described as Schöck Combar. The bars used were made of E-CR (Electrical/Chemical Resistance) glass fiber composite with a diameter of approximately 20 μm and vinyl ester resin. The head anchorage was a fiber reinforced polymer mortar. The anchor head was 60 mm long, and the maximum diameter was 30 mm (Figure 3a). In order to compare the mode of failure and tensile strength of GFRP bars, five samples of Φ12 headed bars and five samples of Φ12 straight bars were tested. The bar tests were carried out in accordance with Annex A of the American standard ACI 440.3R-04 [32] and the instruction ISO 10406-1:2015 [33]. Test samples were made by embedding the bar ends in Sikadur-330 epoxy resin filling internally screwed steel pipes (Figure 3b). The head anchorage was at one end of the test bar. The end of the head anchoring was embedded in a steel element with a special flange (Figure 3c) to eliminate clamping forces that could distort the test results. For the tensile test, samples with a multiplicity of 10 (measuring base length 120 mm for bars Φ12) were used. The modulus of elasticity was determined on the basis of strain results based on a 60 mm long measurement base.

Tensile strength *f*_u_, modulus of elasticity *E*_f_, and ultimate strain *ε*_u_ were determined by the procedure in [32] using the following relationships:

(1) $$f_{u}=\frac{F_{u}}{A}$$

(2) $$E_{f}=\frac{F_{1}-F_{2}}{\left(\varepsilon_{1}-\varepsilon_{2}\right)A}$$

(3) $$\varepsilon_{u}=\frac{F_{u}}{E_{f}A}$$

where: *F*_u_, the tensile capacity of FRP bar; *A*, the cross-sectional area of FRP bar; *F*_1_, the tensile load at approximately 50% of the ultimate load capacity; *ε*_1_, the tensile strain at approximately 50% of the ultimate load capacity; *F*_2_, the tensile load at approximately 20% of the ultimate load capacity; *ε*_2_, the tensile strain at approximately 20% of the ultimate load capacity.

**Figure 4:**
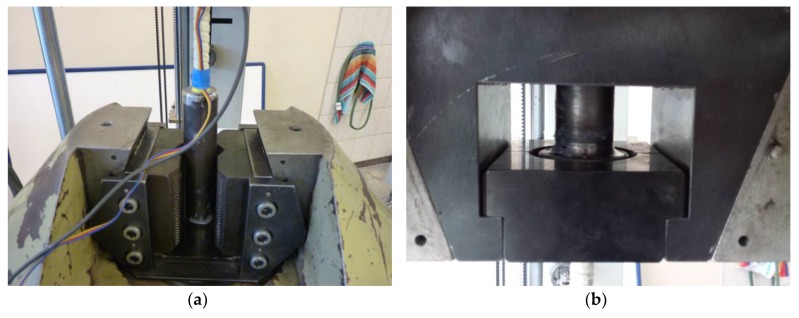
The method of fixing the sample in the testing machine: (**a**) fixing the straight steel pipe; (**b**) fixing of a flanged steel pipe.

Straight bars were stretched in a testing machine using classic jaws used for the tensile test (Figure 4a), steel bars among others. The end in the form of a steel flange was fixed in a special jaw shown in Figure 4b. The use of this type of jaw, with an appropriate wall thickness of the steel tube, eliminated clamping forces that might have distorted the result of the actual anchoring strength.

Based on the data provided by the manufacturer and by Kurth [24], the average tensile strength of the stirrups used was set at *f*_bend_ = 770 MPa and the mean value of modulus of elasticity at *E* = 57 GPa. The stirrups used in this study were bent during manufacture, and the bending diameter was seven times the diameter of the bar (84 mm). The stirrups shaped in the same way were the subject of research by the manufacturer and Kurth in [24]. In this study, the stress-strain characteristics of GFRP stirrups were not experimentally determined, and the values of the tensile strength and modulus of elasticity of GFRP stirrups were taken from the manufacturer’s data, which were consistent with Kurth’s results [24]. 

### 2.3. Instrumentation, Test Setup and Procedure

**Figure 5:**
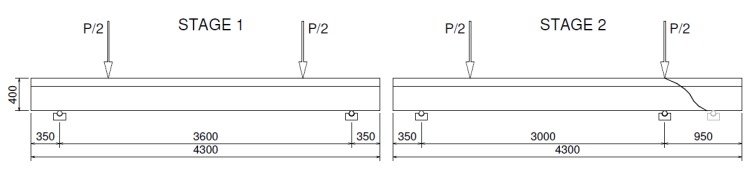
Test setup of two stages. Dimensions in mm.

The examination of each beam consisted of two stages. In the first stage, the beam was subjected to a 4-point bending until one of the support zones was damaged (Figure 5). In the second stage, the support at which damage occurred was moved directly under the force, obtaining a 3-point bending scheme. 

For the first stage, the load causing the appearance of diagonal and perpendicular cracks was determined. The angle of inclination of the compressed concrete strut was also established for each stage. The element was loaded until the appearance of cracks, and the load was then increased at a speed of about 0.2 kN/s, stopping at ca. every 50 kN; each time the crack pattern and the load at which it occurred was monitored. For each beam, strain measurements of concrete were made in the compression zone and the tension bars. Concrete strains were measured using RL300/50 resistivity strain gauges. Strain gauges were glued onto the concrete around the stirrups (on both sides of the web). Beam deflections were recorded in the middle of the span and under concentrated forces using inductive sensors. Displacement of supports was also measured using inductive sensors located next to the support points. The strains of steel bars and GFRP bars were measured with the use of RL120/20 strain gauges. Strain gauges on bars were placed in the middle of the length of the vertical arms of stirrups/bars with head anchoring. Strain gauges were also glued to longitudinal bars in the middle of the span and in cross-sections under applied forces. 

## 3. Experimental Results and Discussion

### 3.1. Rebar Test

**Figure 6:**
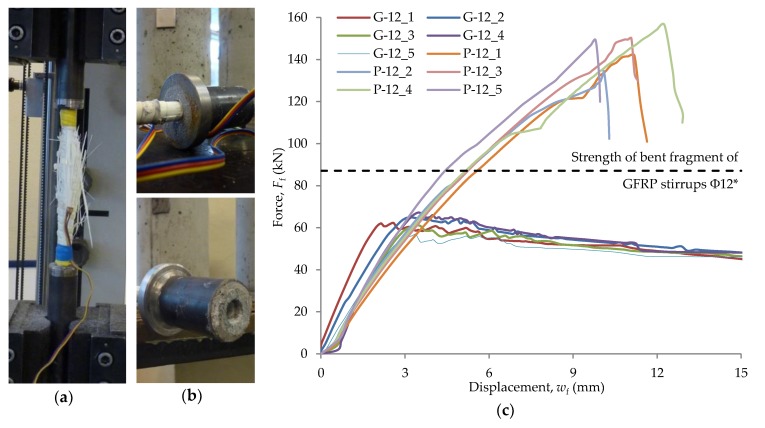
Rebar test results: (**a**) straight bar sample failure; (**b**) headed bar sample failure; (**c**) static equilibrium paths in the tensile test of various types of GFRP bars. * Based on the manufacturer’s data and Kurth’s tests [24].

The failure mode of straight bars was of an explosive nature, and all bars were characterized by linear-elastic behavior over the entire strength range. The average tensile strength of a straight GFRP bar was 1299 MPa with a standard deviation *s* = 69 MPa. The mean modulus of elasticity was 58.1 GPa, with a standard deviation of *s* = 3.4 GPa. The mean value of the ultimate strain was 22.4‰ with a standard deviation *s* = 0.7‰. The damaged sample after the test is shown in Figure 6a, while the static equilibrium paths of the tested bars are shown in Figure 6c.

Headed bars were characterized by linear-elastic behavior until stress initiating the loss of bar adhesion to the anchor head was reached. The failure mode consisted of the slipping of the bar from the anchor head and was radically less violent than bar rupture. Vint tested the anchoring of GFRP headed bars in concrete and came to similar conclusions [21]. A relatively high value of bar displacement relative to the anchor head was achieved, at which the sample was still able to carry the load. The average value of the tensile strength of the GFRP headed bar was 545 MPa with a standard deviation *s* = 15 MPa. The average modulus of elasticity was 61.9 GPa, with a standard deviation of *s* = 3.0 GPa. The damaged sample after the test is shown in Figure 6b, while the static equilibrium paths of the tested bars are shown in Figure 6c.

Before the test, the bar diameter was measured with an accuracy of 0.1 mm. The diameter was measured taking into account the ribs, then the thickness of the ribs was measured, and the core diameter of the bar cross-section *d*_f,i_ was calculated. Fifteen diameter measurements were made for each bar over a distance of 200 mm. The average value of the diameter *d*_f_ is given in Table 2. The tensile strength *f*_u_, modulus of elasticity *E*_f_, and ultimate strain *ε*_u_ were determined based on Formulas (1)–(3), and the results are shown in Table 2.

The short-term tensile strength of headed bars was lower than that of straight bars. The strength of headed bars was about 42% of the tensile strength of straight bars. In addition, based on data from the manufacturer, it was found that the strength of headed bars constituted about 71% of the strength of the bent stirrup section (Figure 6c). The modulus of elasticity for both straight bars and headed bars was similar and amounted to about 60 GPa, which roughly corresponded to the manufacturer’s data, including the research by Kurth [24]. Similarly to the paper [24], where GFRP Φ16 headed bars were tested, the modulus of elasticity obtained as a result of testing headed bars was slightly larger than that obtained in the tensile test of bars without anchoring. The average ultimate strain recorded for the tensile test of straight bars was about 22.4‰, which corresponded to an elongation of about 1.3 mm (on a measuring base of 60 mm). In the case of stirrups, based on the assumption of linear-elastic behavior in the tensile test and the value of the average tensile strength and modulus of elasticity declared by the manufacturer, the estimated ultimate strain was about 13.5‰, which corresponded to an elongation of about 0.8 mm (based on measurement equal to 60 mm). The head length of 60 mm introduced the possibility of transferring the force of almost equal tensile strength of the anchorage at an elongation of about 2.5 mm. It could be concluded that a single bar with a head anchorage had a lower tensile strength than a straight bar or stirrup. In the case of a support zone reinforced with headed bars, greater stress redistribution could be expected than in the case of the corresponding reinforcement in the form of frame stirrups.

### 3.2. Beam Test

**Figure 7:**
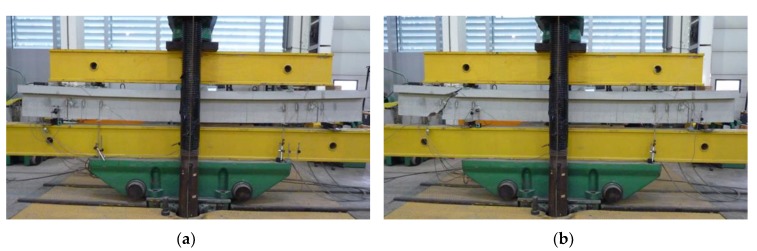
B-1 beam on the test stand during: (a) first stage of the test; (**b**) second stage of the test.

Each beam was tested in two stages in order to obtain results from eight support zones. Figure 7 presents an example on the B-1 beam on the test stand during the first and second stages of the test. 

The subject of the analysis was both the general behavior of concrete elements reinforced with composite bars and the shear issue, which was the main subject of this study. This article presents the results in terms of bearing capacity and failure mode, static equilibrium paths, and the strains of the reinforcement of the support zone. Complete results of leading and accompanying studies were presented by Drzazga in [27]. 

#### 3.2.1. Failure Load and Mode of Failure

**Figure 8:**
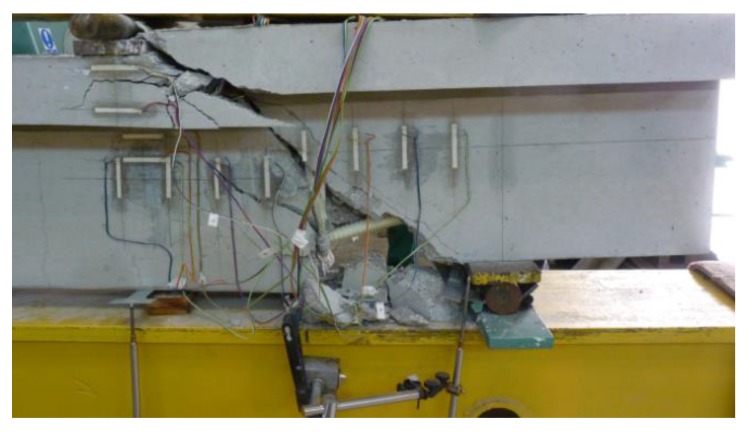
Illustration of the failure mode of a support zone (B-2_160).

All beams were designed in order to obtain the failure in the support zone. All the support zones were damaged as a result of the failure of the transverse reinforcement cut through a diagonal crack (shear–tension failure) (Figure 8).

**Figure 9:**
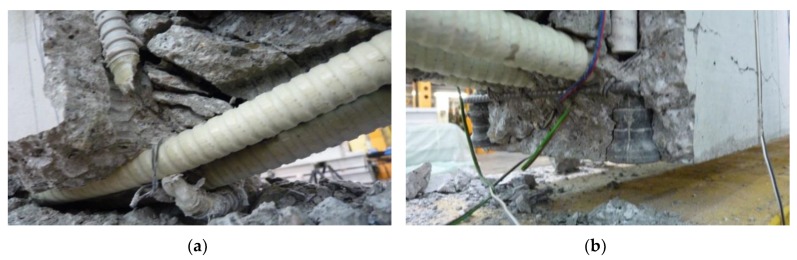
Failure mode of support zones: (**a**) reinforced with GFRP stirrups; (**b**) reinforced with GFRP headed bars.

**Figure 10:**
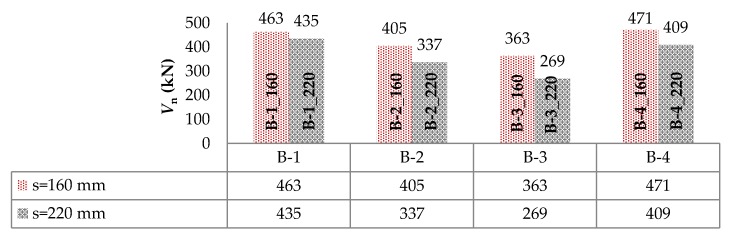
Comparison of the load-bearing capacity of support zones due to shear reinforcement spacing.

The failure of the support zones reinforced with GFRP stirrups was of a violent nature and occurred with the rupture of the stirrup near the bend (Figure 9a). The support zones reinforced with headed bars were marked by a less violent nature of failure, which was associated with the slipping of the bar out of the anchor head (Figure 9b). Figure 10 shows a comparison of the load capacity of individual support zones due to shear reinforcement spacing.

**Figure 11:**
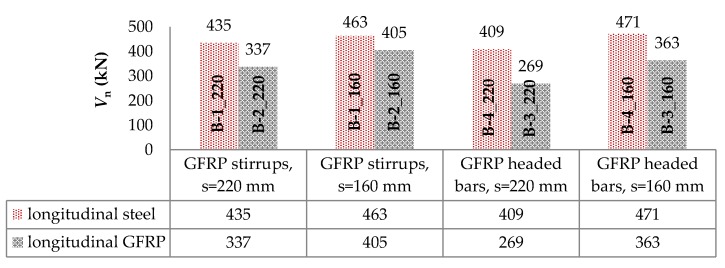
Comparison of the load-bearing capacity of support zones by type of longitudinal reinforcement.

The compaction of shear reinforcement, from 220 mm to 160 mm, resulted for the B-1, B-2, B-3, and B-4 beams in increasing the load capacity by approximately 6%, 20%, 35%, and 15%, respectively. The increase in load capacity was clearly greater for beams with longitudinal composite reinforcement. It can also be observed that using a larger number of bars for shear stress transfer, which being cut by a diagonal crack accounted for the contribution of transverse reinforcement in shear capacity, resulted in a greater increase in load capacity for support zones reinforced with headed bars than with stirrups. Along with the increase in load, an increase in the strains of headed bars was recorded up to the initiation of the slipping of the bars (furthest from the top of the crack) out of their anchor heads. Along with the slipping of the bar out of the anchor head, the level of strains on this bar stabilized and the stress was redistributed, which allowed for a more regular distribution of headed bars’ strains than in the case of stirrups. Figure 11 shows a comparison of the load capacity of individual support zones by type of longitudinal reinforcement. Support zones longitudinally reinforced with steel bars were marked by a higher load carrying capacity than those longitudinally reinforced with GFRP bars. This confirmed the current conclusions from studies by other authors, including [10,11,12,13,14,15]. For GFRP stirrups spaced 220 mm and 160 mm, the use of longitudinal steel reinforcement resulted in an increase in load capacity of approximately 29% and 14%, respectively. In the case of GFRP headed bars spaced 220 mm and 160 mm, the use of longitudinal steel reinforcement resulted in an increase in load capacity by approximately 52% and 30%, respectively. 

**Figure 12:**
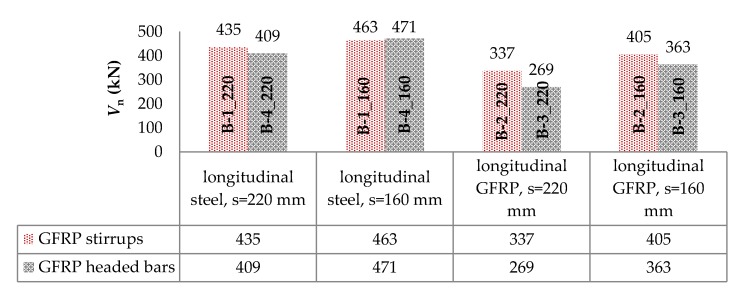
Comparison of the load-bearing capacity of support zones by type of shear reinforcement.

Figure 12 shows a comparison of the load capacity of individual support zones by type of shear reinforcement.

Support zones reinforced with GFRP stirrups were characterized by a higher load carrying capacity than those reinforced with GFRP headed bars. The exception were the support zones B-1_160 and B-4_160, where the support zone with headed bars had a carrying capacity of about 2% higher. This was caused, among other things, by the greater ability of headed bars to redistribute strains (destructive crack cut two pairs of bars). In the case of longitudinal steel reinforcement and shear reinforcement spaced 220 mm, the load capacity of the supporting zone reinforced with stirrups was greater by about 6% than that reinforced with headed bars. Similar values of bearing capacity of support zones longitudinally reinforced with steel bars may result from the high axial stiffness of longitudinal reinforcement. As a consequence, a substantial part of the shear capacity was the bearing capacity of concrete and longitudinal reinforcement. In the case of longitudinal GFRP reinforcement, this increase, for the spacing *s* = 220 mm and *s* = 160 mm, was about 25% and 12%, respectively.

#### 3.2.2. Load Deflection Behavior

The deflection of the research elements was determined on the basis of the results recorded by five induction sensors with an accuracy of 0.001 mm. Two sensors were located at the supporting plates, two directly under the forces and one in the middle of the span. Recording of displacement values took place continuously.

Longitudinally reinforced beams with fiber glass bars (B-2 and B-3) were characterized by higher deflection values at individual load levels than longitudinally reinforced beams with steel bars (B-1 and B-4). This was due to the fact that in the case of beams with longitudinal steel reinforcement, five longitudinal bars were used in the span zone. In addition, the greater axial stiffness of steel reinforcement (as compared to GFRP) resulted in greater bending stiffness of the reinforced concrete element, which was particularly evident in the cracked phase, where the stiffness of an element was largely determined by the modulus of elasticity of longitudinal reinforcement, and this modulus was more than three times smaller for GFRP bars than for steel bars.

**Figure 13:**
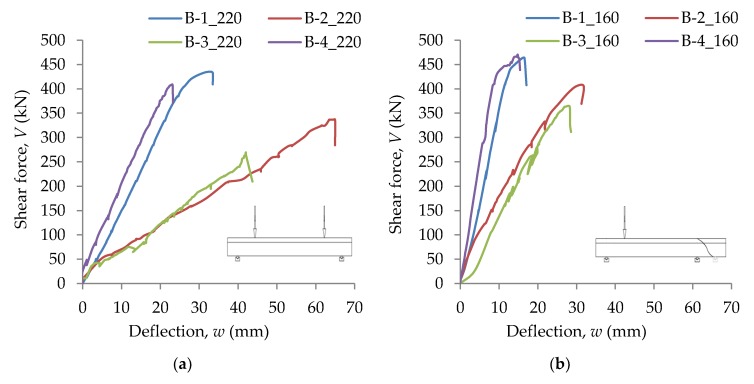
Static equilibrium paths of beams: (**a**) Stage 1; (**b**) Stage 2.

In Figure 13a,b, static equilibrium paths for B-1, B-2, B-3, and B-4 beams are shown for Stages 1 and 2, respectively. In the case of Stage 1, the displacement *w* was recorded in the middle of the span, while for Stage 2, under concentrated force.

#### 3.2.3. Strains in GFRP Stirrups and Headed Bars

**Figure 14:**
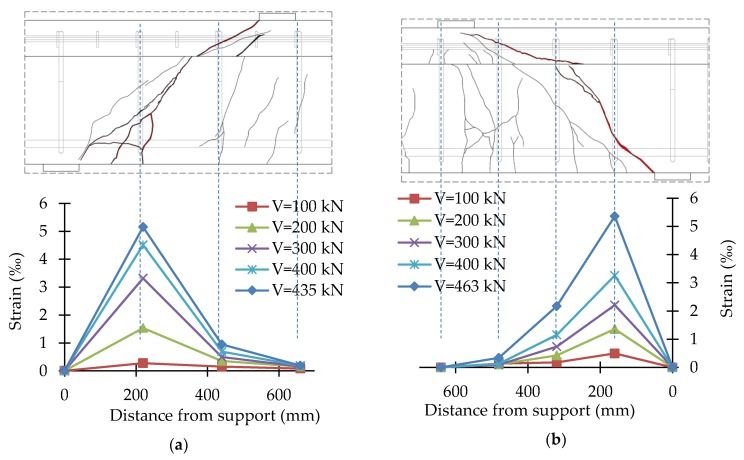
Distribution of strains of the B-1 beam support zone: (**a**) B-1_220; (**b**) B-1_160.

**Figure 15:**
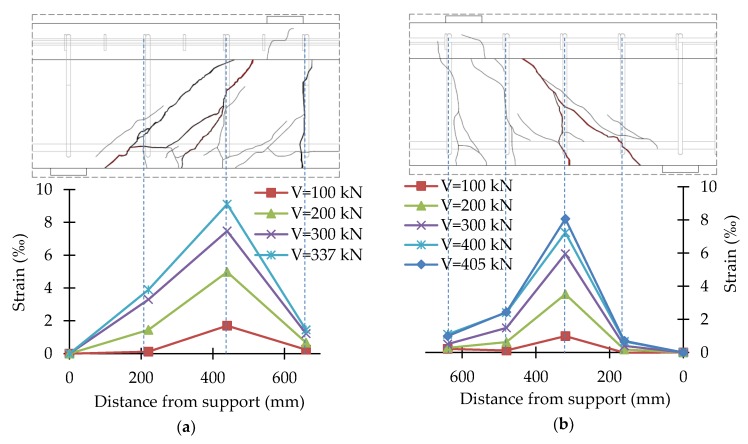
Distribution of strains of the B-2 beam support zone: (**a**) B-2_220; (**b**) B-2_160.

**Figure 16:**
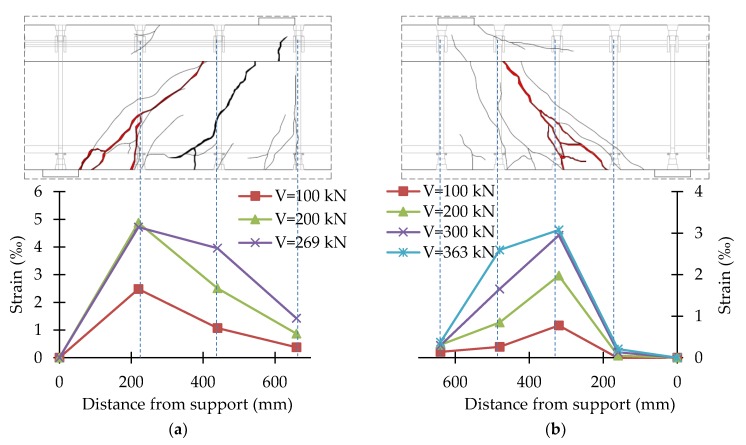
Distribution of strains of the B-3 beam support zone: (**a**) B-3_220; (**b**) B-3_160.

**Figure 17:**
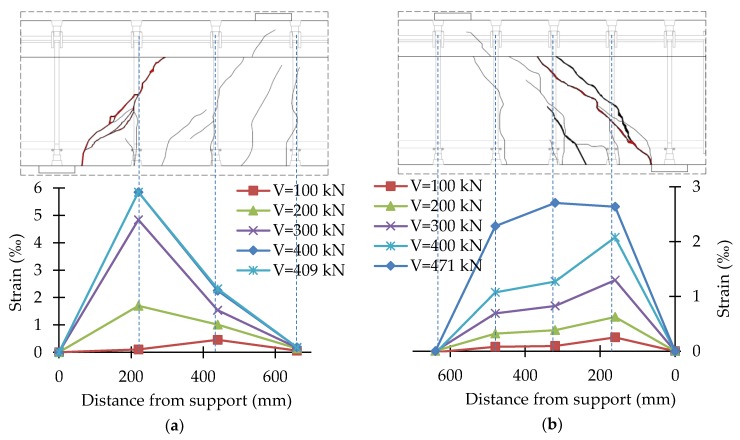
Distribution of strains of the B-4 beam support zone: (**a**) B-4_220; (**b**) B-4_160.

In the course of the tests, the strains of stirrups and headed bars were recorded continuously. Strains were recorded in the central part of the vertical straight segment of stirrups and in the middle of headed bars. The test results were presented in the form of shear reinforcement strains for different load levels together with the crack pattern of the support zone. The strain values given were the average of two vertical straight sections of stirrups or headed bars. Figure 14, Figure 15, Figure 16 and Figure 17 show the distribution of bars strains for B-1, B-2, B-3, and B-4 beam support zones, for different load levels.

In the case of support zones reinforced with GFRP stirrups, along with the increase in load, the increase in stirrup strains was recorded, almost proportionally, until failure. Strains of headed bars increased almost in proportion until strains initiating the loss of bar adhesion to the anchor head were reached. If one pair of headed bars was cut, failure occurred as a result of the slipping of the bar out of the anchor head. If two pairs of bars were cut, along with an increase in load, an increase in the strains of bars with head anchoring was recorded up to the initiation of the slipping of the bars (furthest from the top of the crack) out of the anchor heads. Along with the slipping of the bar from the anchor head, the level of strains on this bar stabilized, and stress was redistributed, which allowed a more regular distribution of strains in the case of headed bars than in the case of stirrups.

For the B-1 beam, the maximum strains recorded on the straight section of stirrups were 5.15‰ (B-1_220) and 5.37‰ (B-1_160). For the B-2 beam, the maximum strains recorded on the straight section of stirrups were 9.10‰ (B-2_220) and 8.07‰ (B-2_160). The difference in the maximum strains recorded may be due to the fact that the strain gauges were glued to the middle of the vertical sections of stirrups. In the case of B-1_220 and B-1_160, the destructive diagonal crack cut the stirrup slightly above the bend. In the case of B-2_220 and B-2_160, the destructive diagonal crack cut the stirrup much closer to the central part of the vertical sections.

For the B-3 beam, the maximum strains recorded on bars with head anchoring were 4.87‰ (B-3_220) and 3.08‰ (B-3_160). For the B-4 beam, the maximum strains recorded on the headed bars were 5.85‰ (B-4_220) and 2.64‰ (B-4_160). Maximum strains in the case of the more heavily reinforced support zone were much smaller than in the case of the less heavily reinforced support zone, as during the tests on a less heavily reinforced support zone, the bars of a more heavily reinforced support zone were also deformed. As a result of the load in Stage 1, the process of bars slipping from anchor heads was initiated.

## 4. Conclusions

The article presented experimental tests of a new type of composite bar that was used as shear reinforcement for concrete beams. The results of the experimental studies of bar tests and short support zones reinforced with GFRP stirrups or GFRP headed bars were presented. On the basis of the experimental studies, the following final conclusions were formulated:

(1) The failure mode of straight bars Φ12 GFRP (Schöck Combar) had an explosive character, and all bars were characterized by linear-elastic behavior over the entire strength range. Based on the manufacturer’s data and research, including Kurth’s research [24], it was found that a similar failure mode accompanied the attempt to stretch the stirrups, where the section in the vicinity of the bend ruptured. GFRP headed bars were characterized by linear-elastic behavior until stresses initiating the loss of bar adhesion to the anchor head were reached. The failure mode consisted of the slipping of the bar from the anchor head and had a radically less violent character than failure by bar rupture. A relatively high value of bar displacement relative to the anchor head was achieved, at which the sample was still able to carry the load.

(2) GFRP headed bars could be used as shear reinforcement. The failure mode of under reinforced support zones consisted in the bar slipping out of the anchor head.

(3) The immediate force initiating the failure of a GFRP headed bar was smaller than the strength of the bent fragment of GFRP stirrups. In the case of a support zone reinforced with headed bars, a more regular distribution of strains could be expected than in the case of analogous reinforcement in the form of frame stirrups, which was confirmed by tests on support zones of beams in a natural scale.

(4) All eight support zones were damaged as a result of the failure of the transverse reinforcement cut by a diagonal crack. The failure of support zones reinforced with GFRP stirrups was violent in its nature and occurred along with the rupture of the stirrup near the bend. Support zones reinforced with headed bars had a less violent mode of failure.

(5) The change of shear reinforcement spacing from 220 mm to 160 mm resulted in an increase in load bearing capacity. The increase in load bearing capacity was clearly greater for beams with longitudinal composite reinforcement. In addition, a greater increase in load bearing capacity was observed for shear zones reinforced with headed bars than for shear zones reinforced with stirrups. Using a larger number of bars cut by a diagonal crack (the contribution of transverse reinforcement in shear capacity) resulted in a greater increase in load bearing capacity for support zones reinforced with headed bars than with stirrups, which indicated a more regular distribution of stress on individual bars cut by a diagonal crack.

(6) The presented research was a new contribution to the experimental study of support zones reinforced with FRP bars. GFRP headed bars could be an alternative for GFRP stirrups and could be used in concrete elements in facilities particularly exposed to aggressive environments and in facilities whose proper functioning is dependent, among other things, on the electromagnetic neutrality of the construction elements. Nevertheless, continuing research in the field of shear behavior of concrete beams reinforced with different type of FRP reinforcement is necessary, both support zones with low and high shear slenderness.

## Figures and Tables

**Table 1:** Reinforcement details of the support zones of tested beams.

Beam Support Zone	Tensile Longitudinal Reinforcement (Shear Zone)			Shear Reinforcement			
	Diameter, ∅_L_	Reinforcement Type	$\rho_{f}=\frac{A_{fL}}{b_{w}d}$	Diameter, ∅_w_	Reinforcement Type	Spacing, *s*	$\rho_{fw}=\frac{A_{w}}{b_{w}s}$
	(mm)	(-)	(%)	(mm)	(-)	(mm)	(%)
B-1_160	20	Steel	1.37	12	GFRP stirrups	160	0.71
B-1_220						220	0.51
B-2_160	20	GFRP	1.37	12	GFRP stirrups	160	0.71
B-2_220						220	0.51
B-3_160	20	GFRP	1.37	12	GFRP headed bars	160	0.71
B-3_220						220	0.51
B-4_160	20	Steel	1.37	12	GFRP headed bars	160	0.71
B-4_220						220	0.51

**Table 2:** Samples details and test results.

Type of Bar	Sample Number	*d*_f_	*F*_u_	*f*_u_	*E*_f_	*ε*_u_
		(mm)	(kN)	(MPa)	(GPa)	(‰)
Straight bars	P-12_1	11.8	141.9	1298	57.8	22.4
	P-12_2	12.0	134.4	1188	53.3	22.3
	P-12_3	12.1	150.1	1305	61.3	21.3
	P-12_4	11.9	156.4	1406	62.4	22.5
	P-12_5	12.1	149.2	1298	55.6	23.3
	Average:	12.0	146.4	1299	58.1	22.4
Headed bars	G-12_1	12.1	62.3	542	65.8	-
	G-12_2	12.2	64.9	555	63.1	-
	G-12_3	12.0	61.2	541	63.5	-
	G-12_4	12.3	67.2	566	57.7	-
	G-12_5	11.8	56.9	520	59.3	-
	Average:	12.1	62.5	545	61.9	-
